# Buried Penis: A Rare Cause of Lower Urinary Tract Symptoms in the Pediatric Population

**DOI:** 10.7759/cureus.42381

**Published:** 2023-07-24

**Authors:** Marta Caldas, Mariana S Pedro, Teresa Magalhães, Mariana Viegas, Anabela Bicho

**Affiliations:** 1 Pediatrics, Centro Hospitalar do Oeste (Western Hospital Center), Caldas da Rainha, PRT

**Keywords:** pediatrics, penis and scrotum, micropenis, lower urinary tract symptoms, penile disease

## Abstract

A buried penis is a probably underdiagnosed entity. It is defined as a penis of normal size that appears to be smaller than expected due to concealment within the pubic tissue. This case report explores the presentation of a 12-month-old male infant with exuberant ballooning of the prepuce during micturition, requiring manual expression of urine for the duration of two months prior to presentation. The penis was not visible above the skin level, with only the glands covered by prepuce protruding. However, the penis could be exposed when holding the base of the penis, revealing a regular-sized penis. The clinical diagnosis of a buried penis with megaprepuce was assumed, and the patient was referred to the pediatric surgery department for further management. Corrective surgery was performed nine months later with excellent cosmetic and functional results. The buried penis has a typical appearance with a partially visible or completely invisible penis, with only the glans covered by prepuce protruding, and it can be completely asymptomatic or cause micturition difficulties, sexual dysfunction, and recurrent urinary tract infections or balanitis. The diagnosis is clinical and the treatment is surgical, although the surgical approach is controversial.

## Introduction

A buried penis is a rare and probably underdiagnosed entity [1]. It is defined as a penis of normal size that appears to be smaller than expected due to concealment within the pubic tissue [1]. The etiology of this entity is not completely understood, but it is suggested that it is associated with a dysplastic dartos fascia, decreased ventral skin, excessive prepubic fat, and lack of attachment of the skin to the penile shaft [2,3]. The diagnosis is clinical, based on the characteristic appearance and physical examination, and it can be associated with symptoms of urinary retention [2]. Urinary retention is a general term referring to storage and/or voiding disturbances, which is more frequent in adults [4]. There are multiple etiologies, including urinary tract infections, anatomical anomalies, and detrusor overactivity [4]. A careful anamnesis and physical examination with evaluation of the abdomen, genitals, and perineum is essential to establish the diagnosis. Genital anatomical anomalies, including inguinal hernias, hypospadias or epispadias, micropenis, and buried penis, are varied and a cause of preoccupation for parents of affected individuals.

## Case presentation

**Figure 1:**
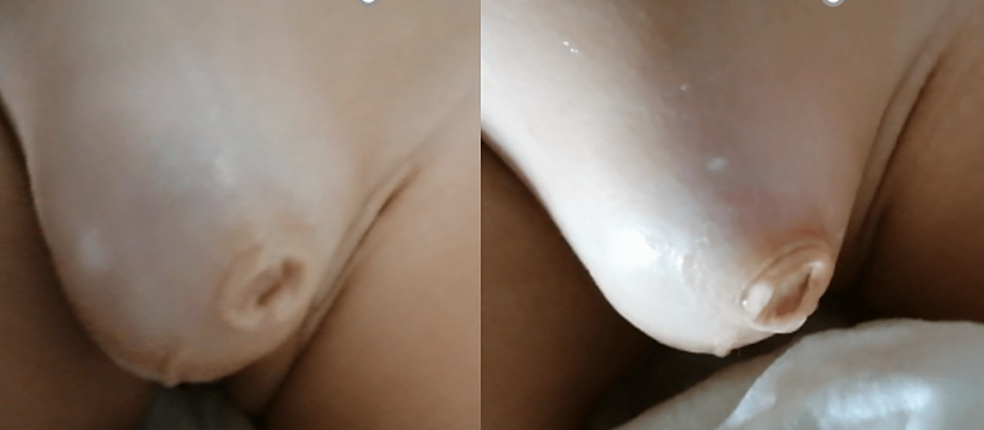
Ballooning of the prepuce during micturition in a buried penis with megaprepuce.

**Figure 2:**
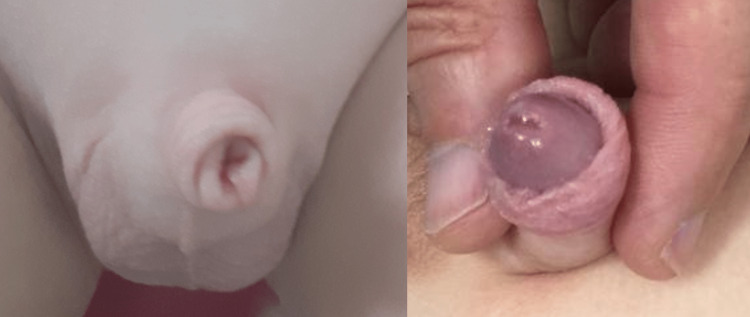
A: Buried penis with megaprepuce with a typical “volcano-like” appearance. B: Normal-sized penis for age exposed by applying finger pressure on the opposite sides of the shaft base.

A 12-month-old male infant presented with exuberant ballooning of the prepuce during micturition, requiring manual expression of urine (Figure 1). The voiding difficulties were noticed two months prior and progressively increased in frequency. The patient was healthy; had no previous history of urinary tract infections, balanitis, or surgeries; and was growing within the 50th percentile based on the World Health Organization Child Growth Standards. Upon physical observation, the penis was not visible above the skin level, with only the glans covered by prepuce protruding, resulting in a “volcano-like” appearance (Figure 2). The penis was exposed after applying pressure on both sides of the base of the penile shaft, retracting the prepuce and penile skin and revealing a regular-sized penis, excluding the diagnosis of a micropenis (Figure 2). The patient did not appear to have phimosis, hydrocele, or inguinal hernia, and the testicles were in the scrotal sac. The clinical presentation was compatible with the diagnosis of a buried penis with megaprepuce, no further studies were performed, and the patient was referred to the pediatric surgery department for further management. Corrective surgery was performed nine months later. The procedure included degloving the penile shaft, releasing the adhesions around the corpus cavernosum, fixating Buck’s fascia, removing the redundant prepuce, and reconstructing the penopubic and penoscrotal angles. There were no postoperative complications, and the procedure had excellent cosmetic and functional results.

## Discussion

A hidden penis is defined as a penis of normal size that appears to be smaller than expected and can be classified as entrapped, webbed, concealed, or buried [1,5]. An entrapped penis is the result of scarring secondary to circumcision [1,5]. A webbed penis is caused by the disappearance of the penoscrotal angle associated with an extension of the skin between the scrotum and the distal shaft of the penis [1,5]. A concealed penis is characterized by insufficient penile skin or inelasticity of the dartos fascia [1,5]. A buried penis occurs when there is concealment within the pubic tissue associated with poor skin fixation at the base of the penis [1,5]. It can be associated with congenital megaprepuce, which is characterized by the presence of extensive redundant prepuce and, consequently, ballooning of the excessive skin, as was observed in this case [2]. 

The typical appearance of a buried penis with megaprepuce is a partially visible or completely invisible penis, with only the glans covered by prepuce protruding [2]. The penis has appropriate dimensions when it is exposed, by applying pressure on opposite sides of the shaft base [2]. This characteristic appearance is similar to our patient's. This entity generally is asymptomatic but can be associated with functional problems, such as voiding difficulties, ballooning of the prepuce, or urine spraying, as was presented in this case [1,2,3]. It can also manifest as pain during micturition and recurrent urinary tract infections or balanitis [1,2,3]. In adolescents, it can also lead to sexual dysfunction and psychological damage, such as anxiety and depression, impaired relationships, and lowered self-esteem [1,3].

The treatment for a buried penis should aim to restore an aesthetic and functional penis [1]. Surgical correction is the gold-standard treatment, but there is no consensus on patient eligibility and timing of the surgery [2,3]. Eroglu et al. defended deterring surgery until puberty is completed, as the buried penis has a tendency to improve with growth and pubertal development [6]. Other authors advise early surgical correction in order to resolve clinical manifestations if present, avoid the negative psychological impact during puberty, and reduce parental anxiety by improving cosmetic appearance [5]. Moreover surgical repair is associated with less complications in infancy, when compared to adolescents and adults [5,7]. The extensive diversity of therapeutic approaches reflects the different perceptions of etiology, and the treatment must be tailored to the individual [1]. There is no single optimal operative technique that has been capable of meeting all patient needs [1]. The most commonly described surgical procedure includes degloving of the penis, excision of the dysplastic dartos attachments, excision of the redundant inner prepuce, and recreation of the penopubic and penoscrotal angles, as was performed in this case [1,2,3,5]. However, the surgical technique used for the dissection of the dartos fascia and fixation of the penile skin to reinforce the penoscrotal and penopubic angles and the decision to remove the excess fat and to perform skin grafting differ according to the center [1,5,7]. 

The complications are mostly minor and temporary and resolve with conservative measures [2,3,5]. The most common complications are hematoma, mild bleeding, penile edema, dehiscence, and superficial infection, which can be resolved by applying ice and administering antibiotics [5,8]. The cosmetic and functional outcome of the surgery is good [2,3]. There is a low recurrence rate of the symptoms, and most patients and/or their families report marked improvement in hygiene and appearance of the penis [5,7,8]. There is a low rate of recurrence and complications that require a new surgical intervention [5,8]. In this case, there were no complications, there was complete resolution of the symptoms, and the parents were satisfied with the cosmetic result.

An important differential diagnosis is the presence a true micropenis, in which the length of the penis is at least two and a half standard deviations below for the patient’s age, contrarily to what was observed in this case [2]. The micropenis is caused by hormonal defects, usually due to a decrease in testosterone, and can be associated with other conditions, such as hypothalamus tumors and genetic syndromes, and the first-line treatment is medical, i.e., by replacing the deficient hormones [2].

## Conclusions

A buried penis is a rare and probably underdiagnosed entity. It has a typical presentation and appearance that should be recognized and identified. Although the penis is covered by excessive skin and prepubic tissue, it becomes visible and has a normal size and characteristics after applying pressure on the base of the penis. As such, it is important to do a thorough physical examination with a careful observation of the penis in order to differentiate from other anatomical anomalies, such as inguinal hernias, hypospadias or epispadias, and micropenis. It is essential to differentiate a buried penis and micropenis as these two entities have distinct etiologies, diagnostic approaches, and treatments. 

This case serves as an important reminder to consider a buried penis in the differential diagnosis when pediatric patients are presenting with lower urinary tract symptoms.
